# Hepatic alveolar echinococcosis with chest wall metastasis: a case report

**DOI:** 10.3389/fmed.2025.1538839

**Published:** 2025-04-15

**Authors:** Meng-Zhao Xu, Fei Ke, Jin-Ping Chai, Ji-De A, Lin-Xun Liu

**Affiliations:** ^1^The Graduate School, Qinghai University, Xining, China; ^2^Department of Internal Medicine-Cardiovascular, Qinghai Provincial People’s Hospital, Xining, China; ^3^Department of Hepatic Hydatidosis, Qinghai Provincial People’s Hospital, Xining, China; ^4^Department of General Surgery, Qinghai Provincial People’s Hospital, Xining, China

**Keywords:** alveolar echinococcosis, liver, chest wall, surgical treatment, case report

## Abstract

Alveolar echinococcosis (AE), a zoonotic parasitic disease caused by *Echinococcus multilocularis* infection, predominantly colonizes the liver and may metastasize to the lungs or brain in advanced stages. Involvement of extrapulmonary sites such as the chest wall or subcutaneous tissues is exceedingly rare, even in endemic regions. The nonspecific clinical manifestations and imaging features of chest wall AE pose diagnostic challenges, necessitating histopathological confirmation. We present a case of a 48-year-old female admitted with a chief complaint of a right supra-mammary mass persisting for over 1 year. Imaging studies revealed a cystic lesion in the right chest wall and a hypodense hepatic lesion in the right lobe, suggestive of hydatid disease. The patient underwent combined hepatic segmentectomy and chest wall mass resection under general anesthesia. Histopathological examination confirmed AE infection in both hepatic and anterior chest wall specimens. The patient achieved complete recovery with no postoperative complications and was discharged uneventfully. Regular oral albendazole therapy has been maintained for 6 months postoperatively, with no recurrence to date.

## Introduction

Alveolarechinococcosis (AE), a zoonotic parasitic disease caused by larval infection of *Echinococcus multilocularis*, is clinically recognized as “worm cancer” due to its infiltrative growth pattern and high mortality rate (1). This disease is predominantly endemic in high-latitude pastoral regions of the northern hemisphere. In China, elevated prevalence areas include the Qinghai-Tibet Plateau, Xinjiang, and western Sichuan, where transmission correlates closely with human contact with canid hosts (e.g., dogs, foxes) and exposure to contaminated natural environments (2, 3). The adult tapeworm resides in the intestinal tract of definitive hosts (primarily foxes and dogs), with eggs being released into the environment through fecal contamination. Humans become accidental intermediate hosts through ingestion of eggs. Following hepatic implantation, larvae develop infiltrative lesions in the liver and may metastasize via hematogenous spread or direct invasion to distant organs, with common metastatic sites encompassing the lungs, brain, and skeletal system (4, 5). Notably, thoracic wall metastasis represents an exceptionally rare clinical manifestation, rarely reported in clinical literature.

Thoracic wall metastasis of AE is sparsely documented in domestic and international literature. Its imaging manifestations often mimic soft tissue neoplasms, tuberculosis, or cystic lesions, further complicating differential diagnosis and frequently resulting in delayed therapeutic intervention. This case report describes a 48-year-old Tibetan woman from a pastoral area of the Qinghai-Tibet Plateau, who initially presented with a mass above the right breast. Imaging revealed hepatic and chest wall lesions, ultimately pathologically confirmed as metastatic AE involving the chest wall. The uniqueness of this case lies in three aspects: ① Atypical Metastatic Presentation: The chest wall, an exceedingly rare metastatic site, exhibited a well-defined cystic lesion, distinct from the infiltrative features of the primary hepatic lesion. ② Diagnostic Pitfalls: The absence of typical hepatic symptoms and the initial misclassification of the breast mass as a benign BI-RADS category 3 lesion on ultrasound underscore the critical role of multidisciplinary collaboration in differentiating complex parasitic diseases. ③ Therapeutic Implications: While surgical resection combined with antiparasitic agents (e.g., albendazole) remains the cornerstone of AE management, the optimal strategy for complete resection of chest wall metastases requires further exploration.

## Case presentation

A 48-year-old woman from a pastoral area presented to our hospital with a palpable mass superior to the right breast. The patient was found a year ago with no obvious causative factors a swelling above the breast on the right chest wall, about the size of a peanut meter, with intermittent tingling pain, and did not receive formal treatment. Recently, the swelling gradually increased in size, so the patient came to our outpatient clinic. The patient was admitted to our department with “anterior chest wall mass,” and since the onset of the disease, the patient’s mental state was clear, his bowel movements were normal, and he had not lost any significant weight recently.

### Physical examination

Physical examination revealed a solid mass was detected in the direction of 10 o’clock in the upper quadrant of the right breast, about 9.0 × 5.0 cm in size, with no ulceration or oozing on the surface, poorly demarcated from the surrounding area, poor mobility and negative tenderness, with a smooth surface and soft texture, and no fluctuating sensation was detected. No enlarged lymph nodes were detected in the axilla, and abdominal examination showed no obvious abnormalities.

### Laboratory examinations

Initial laboratory tests showed: Red blood cells, 4.53 × 10^12^ cells/L, white blood cells 5.54 × 10^12^ cells/L, hemoglobin 116 g/L, platelet count 264 × 10^9^ cells/L. Liver function: alanine aminotransferase: 9 U/L, aspartate aminotransferase 13 U/L, total bilirubin 7.6 mol/L, direct bilirubin: 1.9 mol/L, albumin 36.5 g/L. Cholinesterase 6491 U/L. Tumor indicators: glycan antigen 15-3: 8.61 U/ml, carcinoembryonic antigen 1.43 ng/ml. Echinococcus IgG Ab 0.83S/CO, negative.

### Imaging examinations

**FIGURE 1:**
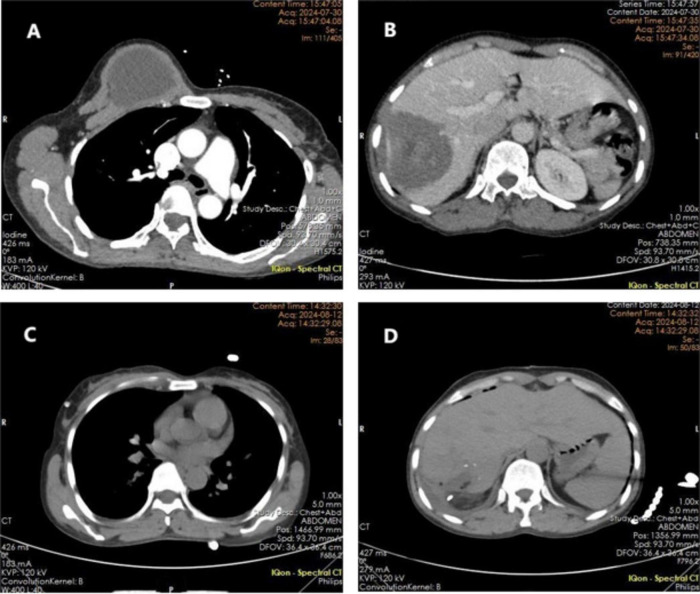
Pre- and post-operative imaging. **(A)** Preoperative computed tomography (CT) image of the chest wall lesion; **(B)** preoperative abdominal CT image of the liver lesion; **(C)** postoperative chest CT image; **(D)** postoperative CT image of the abdomen.

Breast ultrasound: right breast solid hypoechoic mass BI-RADS 3. Computed tomography (CT) of chest and abdomen: cystic density shadow of about 63 × 53 mm in size on the right chest wall, with clear boundary, no obvious abnormal enhancement after enhancement (Figure 1A). The liver is enlarged, and the right lobe of the liver can be seen as a low-density occupancy of about 70 × 67 mm, with patchy high-density shadows and nodular calcifications, and inhomogeneous enhancement in the right lobe of the liver after enhancement (Figure 1B).

### Surgical treatment

Following multidisciplinary consultation, the patient was preoperatively diagnosed with hepatic AE and a right chest wall mass. Preoperative evaluation demonstrated normal cardiopulmonary function and Child-Pugh class A liver function (5 points). On August 6, 2024, the patient underwent combined hepatic segmentectomy and right chest wall mass resection under general anesthesia, performed collaboratively by hepatobiliary and breast surgery teams.

During Phase I surgery conducted by the hepatobiliary team, standard abdominal skin disinfection was performed using povidone-iodine. A reverse L-shaped upper abdominal incision was made to access the peritoneal cavity through layered dissection. Exploratory laparotomy revealed no ascites or metastatic lesions. Hepatic examination identified an ovoid cystic-solid mass in the right lobe with characteristic grayish-white surface features of AE. The lesion demonstrated local tissue adhesion without involvement of the first/second hepatic hilum or inferior vena cava. Remnant liver parenchyma appeared normal in texture. Iodine-soaked gauze was circumferentially placed around the lesion for protective isolation. Parenchymal transection was performed using ultrasonic dissection combined with bipolar electrocautery along a 1.5 cm resection margin. Branches of the portal vein and hepatic artery in segments S5/S6 were ligated and divided. Meticulous dissection of right hepatic vein tributaries ensured complete lesion excision. Hemostasis and bile leak prevention were confirmed through systematic inspection. Major biliary and vascular structures at the resection margin were secured with 4-0 Prolene sutures. Final hemostasis was achieved using argon beam coagulation followed by gelatin sponge application. Two closed suction drains were placed in the subhepatic space and Winslow’s foramen, exteriorized through separate right abdominal incisions. Standard abdominal closure was performed following confirmation of instrument and gauze counts.

**FIGURE 2:**
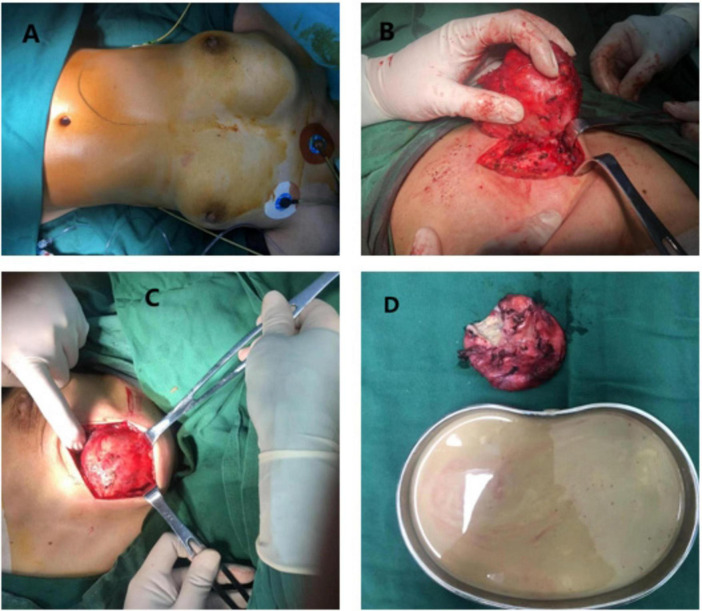
Intraoperative pictures. **(A)** Lesions located above the right breast; **(B,C)** intraoperative chest wall lesions; **(D)** incision of the chest wall lesions.

The patient subsequently underwent immediate resection of the right chest wall mass. Following standard surgical site disinfection and draping, a cystic-solid mass measuring 8.0 × 4.0 cm was palpated between the 11–3 o’clock positions of the right breast, exhibiting well-defined margins and moderate mobility (Figure 2). A Langer’s line incision was made over the mass surface. Sequential dissection through skin, subcutaneous tissue, and bilateral skin flaps revealed the lesion beneath the glandular tissue. The mass was localized deep to the pectoralis major muscle. Surgical access was achieved through intermuscular planes of the pectoralis major to expose the tumor surface. Circumferential dissection along the tumor periphery demonstrated invasion of the pectoralis minor muscle and significant adhesion to parasternal tissues. Adherent parasternal tissues were progressively clamped, divided, and ligated to achieve complete tumor excision. Satellite lesions identified in the intercostal muscles between the 3rd and 4th ribs were managed with electrocauterization. Meticulous hemostasis was achieved prior to layered closure. The residual mammary cavity and pectoralis major muscle were approximated with interrupted sutures. No active bleeding was observed upon final inspection. The excised specimen was visually verified by the patient’s family before submission for histopathological analysis. Postoperative management included hemostatic agents and supportive fluid therapy. The patient was transferred to the ward in stable condition following an uneventful recovery from anesthesia.

**FIGURE 3:**
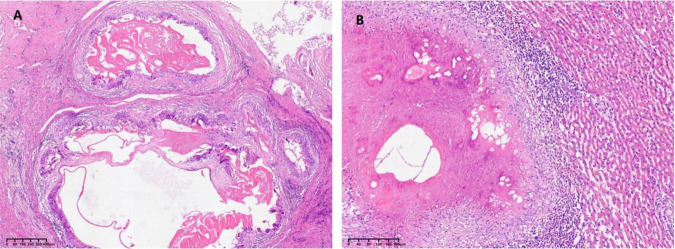
Postoperative pathological images. **(A)** Hematoxylin and eosin (H&E)-stained pathological images of the chest wall lesion; **(B)** Hematoxylin and eosin -stained pathological images of the liver lesion.

The patient exhibited an uneventful postoperative recovery. Histopathological examination of both hepatic and chest wall specimens confirmed the diagnosis of AE (Figure 3). A contrast-enhanced CT scan performed on postoperative day 7 demonstrated: ① Post-resection status of the right chest wall cystic lesion with drainage tube placement, showing no significant fluid or gas accumulation at the surgical site, accompanied by a small right pleural effusion (Figure 1C); ② Status post combined hepatic segmentectomy, with expected postoperative fluid and gas collections in the resection area (Figure 1D). The patient achieved full recovery and was discharged on postoperative day 8.

### Outcome and follow-up

The patient achieved an uneventful postoperative recovery. Oral albendazole therapy was initiated postoperatively, and no recurrence was observed during the 6-month follow-up period.

## Discussion

After infection with *Echinococcus multilocularis* in humans, the larval stage primarily invades the liver via the portal venous system (accounting for over 90% of cases) and may metastasize to other organs (e.g., lungs, brain, bones) via the bloodstream or lymphatic system. Chest wall metastasis in AE is an exceedingly rare clinical phenomenon, predominantly associated with local extension from hepatic primary lesions. The exact incidence of thoracic wall involvement lacks large-scale epidemiological data, with existing studies predominantly based on case reports or regional cohort analyses (6, 7). The underlying mechanisms, diagnostic strategies, and therapeutic approaches for thoracic wall metastasis in AE exhibit distinct particularities. This case, representing the first pathologically confirmed hepatic-origin thoracic wall metastatic AE in our institution, provides critical insights into the clinical management of this rare complication. It further highlights the complexity of AE’s biological behavior and underscores the necessity of multidisciplinary collaborative care.

Metastatic pathways of AE primarily involve direct invasion, hematogenous dissemination, and lymphatic spread. While hepatic lesions may directly extend through the diaphragm to invade the pleural cavity and chest wall, in this case, the hepatic lesion localized in segments S5/S6 of the right hepatic lobe demonstrated no anatomical continuity with the chest wall metastasis. Moreover, CT imaging revealed a well-demarcated cystic lesion in the chest wall, inconsistent with direct invasion. Current evidence suggests hematogenous dissemination as the predominant metastatic mechanism: *Echinococcus multilocularis* vesicles may infiltrate hepatic venous branches, subsequently entering the inferior vena cava–right heart–pulmonary artery circulation to establish distant metastases. However, pulmonary tissue typically functions as a “biological filter,” trapping parasitic emboli. Systemic dissemination to peripheral tissues (e.g., chest wall) requires right-to-left shunting (e.g., patent foramen ovale) to bypass pulmonary filtration (7). Notably, no cardiac structural abnormalities were observed in this patient, suggesting the potential presence of microvascular shunts or lymphatic-vascular interactions contributing to metastasis—a mechanism similarly proposed in cases of extrahepatic metastasis reported by Reuter et al. (8). Furthermore, the rich vascular supply and high metabolic activity of thoracic wall soft tissues may provide a favorable microenvironment for larval colonization (9).

This case initially manifested as a painless chest wall mass, misclassified as a BI-RADS category 3 benign lesion on breast ultrasound, reflecting insufficient clinician awareness of AE metastasis to rare sites. Imaging features of chest wall AE lack specificity: CT may demonstrate unilocular/multilocular cystic lesions, calcifications, or solid components, mimicking soft tissue sarcoma, tuberculous cold abscess, or metastatic carcinoma (10). Notably, the chest wall lesion in this case exhibited a purely cystic appearance, contrasting sharply with the calcified primary hepatic lesion. This discrepancy may correlate with distinct developmental stages—the intrahepatic primary lesion developed calcifications due to chronic inflammation, whereas the chest wall metastasis, in an active growth phase, lacked dystrophic calcification. AE diagnosis requires multimodal integration of imaging, serology, and histopathology. Contrast-enhanced ultrasound (CEUS) has recently proven valuable in differentiating AE from hepatocellular carcinoma: AE typically shows no arterial-phase enhancement with marginal portal-phase enhancement, whereas malignancies often exhibit early hyperenhancement (11). However, CEUS was not performed for the superficial chest wall lesion in this case, potentially delaying differential diagnosis. PET-CT demonstrates superiority in assessing metabolic activity of metastases, yet its sensitivity for cystic lesions remains limited (∼60%), restricting utility in chest wall AE evaluation (12). Serologically, the WHO recommends Em18 antibodies as the primary diagnostic marker due to high specificity (>95%). Importantly, 10%–15% of immunocompetent patients may yield false-negative serology, particularly in early or localized disease (13), necessitating comprehensive diagnostic integration. Histopathological examination remains the gold standard. Postoperative histology in this case revealed pathognomonic germinal layer structures and laminated keratinous membrane. However, preoperative fine-needle aspiration (FNA) biopsy was avoided due to risks of cystic fluid leakage and anaphylaxis (14).

The treatment objectives for AE encompass complete lesion resection and suppression of parasitic activity. In this case, R0 resection was achieved through combined irregular hepatectomy and en bloc excision of the chest wall mass, followed by adjuvant albendazole chemotherapy, consistent with international guideline recommendations (15). Surgical management of chest wall metastasis, however, presents dual challenges: ① Anatomic Complexity: Critical structures such as intercostal nerves and pleural adhesions increase the risk of microscopic residual disease. ② Reconstructive Balance: Post-defect repair necessitates equilibrium between functional preservation and cosmetic outcomes. In this patient, extended resection (incorporating partial intercostal muscles) was performed. No recurrence was observed during the 6-month postoperative follow-up, underscoring the imperative of radical excision.

Long-term postoperative suppressive therapy is crucial for preventing recurrence. Albendazole (10–15 mg/kg/day) should be administered for at least 2 years after surgery (13). In this case, the patient has completed 6 months of postoperative drug therapy with no signs of recurrence to date; however, ongoing monitoring of serum Em18 antibody titers and imaging changes remains essential. For unresectable metastatic AE, recent studies have explored the synergistic effects of targeted therapies (e.g., everolimus for mTOR pathway inhibition) and interventional approaches (e.g., drug-eluting bead embolization). Animal experiments demonstrate that such strategies suppress vesicle proliferation and promote calcification (16), though their clinical efficacy requires validation through large-scale trials.

## Conclusion

Medical professionals should emphasize the accurate diagnosis of AE, especially in rare locations such as the chest wall. Preoperative diagnosis of rare-site lesions enables better assessment of the patient’s condition and facilitates individualized treatment planning, intraoperative strategies, and prevention of postoperative complications. Selecting the appropriate surgical procedure and adjunctive drug therapy ensures effective management and long term follow up, minimizing the risk of recurrence.

## Data Availability

The original contributions presented in this study are included in this article/supplementary material, further inquiries can be directed to the corresponding authors.
